# Polysilyne chains bridged with beryllium lead to flat 2D Dirac materials

**DOI:** 10.1038/s41598-023-40481-2

**Published:** 2023-08-14

**Authors:** Masae Takahashi

**Affiliations:** Department of Physics, Graduate School of Science, Tohoku University, Sendai, 980-8578 Japan

**Keywords:** Chemical bonding, Electronic properties and materials, Two-dimensional materials, Computational science, Two-dimensional materials, Electronic properties and materials

## Abstract

Polysilyne with repeating disilyne units, a silicon analogue of polyacetylene, has a high potential for application to various novel silicon-based electronic devices because of the unique properties of Si=Si units with a smaller HOMO–LUMO energy gap than that of C=C units. However, one-dimensional (1D) polysilyne has not been synthesized yet. Here we propose a planar and air-stable two-dimensional (2D) silicon-based material with one-atom thickness consisting of beryllium-bridged 1D *all-trans* polysilyne, based on the first-principles calculations. The flat structure of 1D polysilyne, which is essential for the air stability of silicon π-electron conjugated systems, is realized by embedding polysilyne in a planar sheet. It was found that the 2D crystal optimized at the rhombus unit cell with the *D*_*2h*_ group symmetry is a silicon-based Dirac semimetal with linear dispersion at the Fermi energy and hosts anisotropic Dirac fermions.

## Introduction

One-dimensional (1D) nanostructures in two-dimensional (2D) materials provide a unique platform to investigate the characteristic 1D properties under a well-defined atomic-scale configuration^1–3^. These novel materials would resolve a long-standing experimental difficulty shared by many 1D polymers that they are hardly fabricated in a crystalline form to yield a sharp X-ray diffraction. Polysilyne with repeating disilyne (HSi≡SiH) units is a silicon analogue of polyacetylene with acetylene (HC≡CH) units. Since the discovery of high electric conductivity in doped polyacetylene^4^, a great attention has been focused on its novel properties as the simplest 1D conjugated polymer^5–9^. On the other hand, its silicon analogue, 1D polysilyne, has not been synthesized yet, while it has a high potential for application to various novel silicon-based electronic devices due to the unique properties of Si=Si units with a smaller HOMO–LUMO energy gap than that of C=C units.

**Figure 1:**
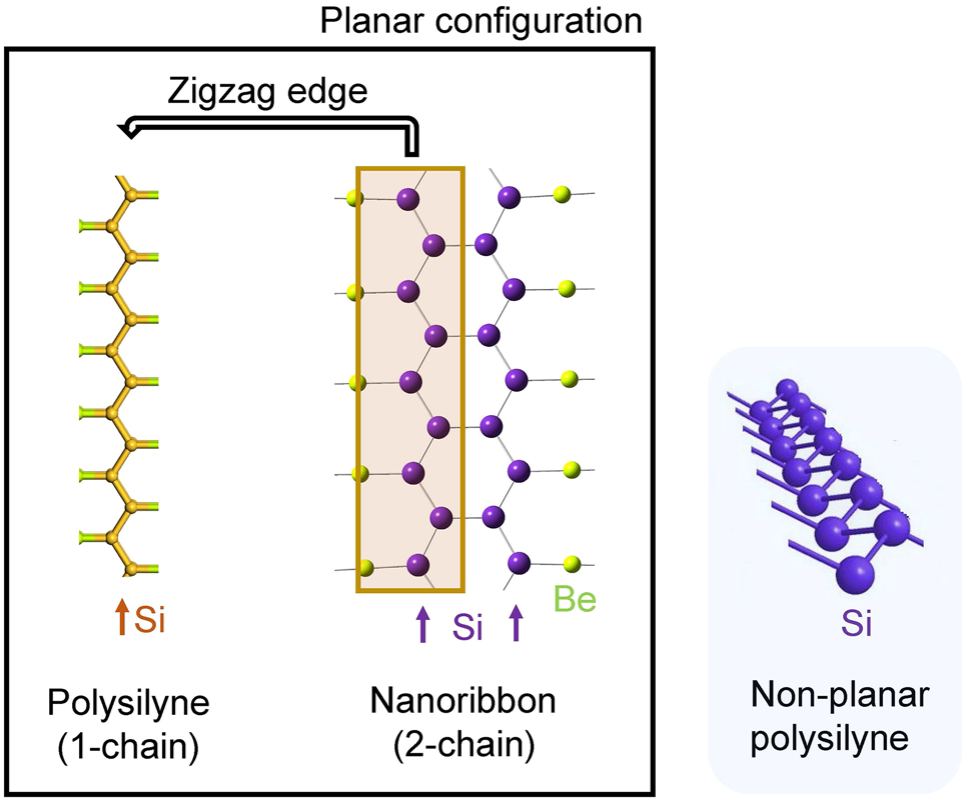
Planar *all-trans* polysilyne and two-chain zigzag silicene nanoribbon (Ref.^24^). Silicon chains are indicated by allows. Non-planar *all-trans* polysilyne is shown on the right for comparison. Images were created using BIOVIA Materials Studio 2018 (https://www.3ds.com/ja/products-services/biovia/products/molecular-modeling-simulation/biovia-materials-studio/) and GaussView 6.0.16, Semichem, Inc. (https://www.hulinks.co.jp/software/chem/gaussview/section02).

It is well known that the flat structure is essential for the π conjugation and the sp^2^ hybridization in the carbon π-electron conjugated systems, such as 1D polyacetylene, 2D graphene, and their building blocks (ethylene and benzene). However, the silicon π-electron conjugated system markedly differs from the carbon counterpart in the structure and stability, despite a similar hybridization occurring in both systems (Refs.^10–14^ and references cited therein). Unlike flat and highly stable graphene, silicene, a silicon equivalent of graphene, is relatively sticky and unstable in the air due to its puckered or crinkled structure^15^. Building blocks of polysilyne (disilene (RSi = SiR)^16^ and tetrasilabuta-1,3-diene^17,18^) have been successfully synthesized by employing the bulky substituent for stable isolation, but they do not exhibit a planar structure, suggesting that a simple extension with these building blocks would not provide planar 1D polysilyne (Fig. 1). On the other hand, by using the electron donation into the silicon π-electron system instead of bulky substituent, linear disilyne, and planar polysilicon chains and hexagons were obtained by our first-principle calculations^19–21^. Experimentally, disilenyllithium (a silicon analogue of vinyllithium) where lithium atoms act as a strong electron donor^22^, was reported to have a reduced twisting angle between two Si–Si–Si planes, although it is still not perfectly planar. Based on these studies on the electron-donated silicon π-electron conjugated systems, we have succeeded in designing a flat building block for flat silicene^23^. Furthermore, we have designed a flat 2-chain zigzag silicene nanoribbon in a flat 2D sheet, where silicene nanoribbons are bridged with beryllium atom (Fig. 1)^24^. These findings have motivated us to design *all-trans* 1D polysilyne in a 2D sheet with a planar configuration, because *all-trans* 1D polysilyne forms an edge of zigzag silicene nanoribbon (Fig. 1).

In this article, we report designing of *all-trans* 1D polysilyne with a planar configuration embedded in a 2D sheet and its unique electronic properties. 1D-polysilyne chains are connected with beryllium atoms to form a 2D sheet, as in silicene nanoribbons embedded in a 2D sheet^24^. We found that the designed polysilyne is a silicon-based Dirac semimetal^25–28^ with linear dispersion at the Fermi energy and hosts anisotropic Dirac fermions. In contract to graphene and many other 2D compounds with an isotropic Dirac cone, designed polysilyne possesses an anisotropic Dirac cone to induce the anisotropic carrier mobility, useful for realization of direction-dependent quantum devices.

## Results and discussion

### *All-trans* polysilyne in 2D sheet with planar configuration

**Figure 2:**
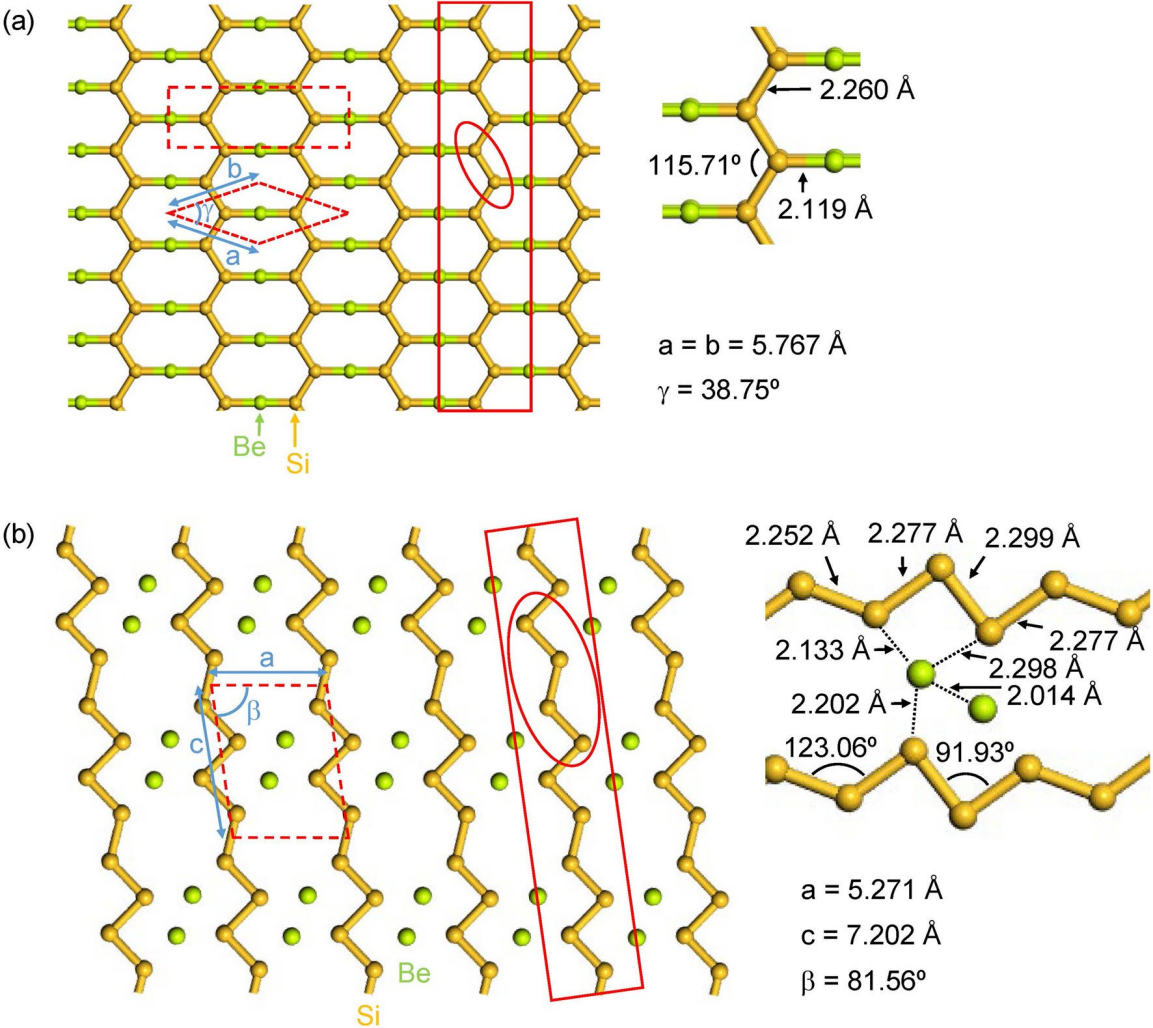
Optimized 2D crystal structures of Be-bridged 1D *all-trans* polysilyne chains, **1a** (**a**) and **1b** (**b**). Polysilyne chains are indicated by red solid rectangles. Silicon and beryllium atoms are shown by gold and green color, respectively. Red dashed lines indicate the primitive and conventional unit cells of the 2D crystal. Red circle indicates the repeating Si unit in the 1D chains. Atomic coordinates are under the group symmetry of *D*_*2h*_ (**1a**) and *C*_*2h*_ (**1b**), respectively. Vacuum spacing of 45 Å was set perpendicular to the sheet. Images were created using BIOVIA Materials Studio 2018 (https://www.3ds.com/ja/products-services/biovia/products/molecular-modeling-simulation/biovia-materials-studio/).

**Figure 3:**
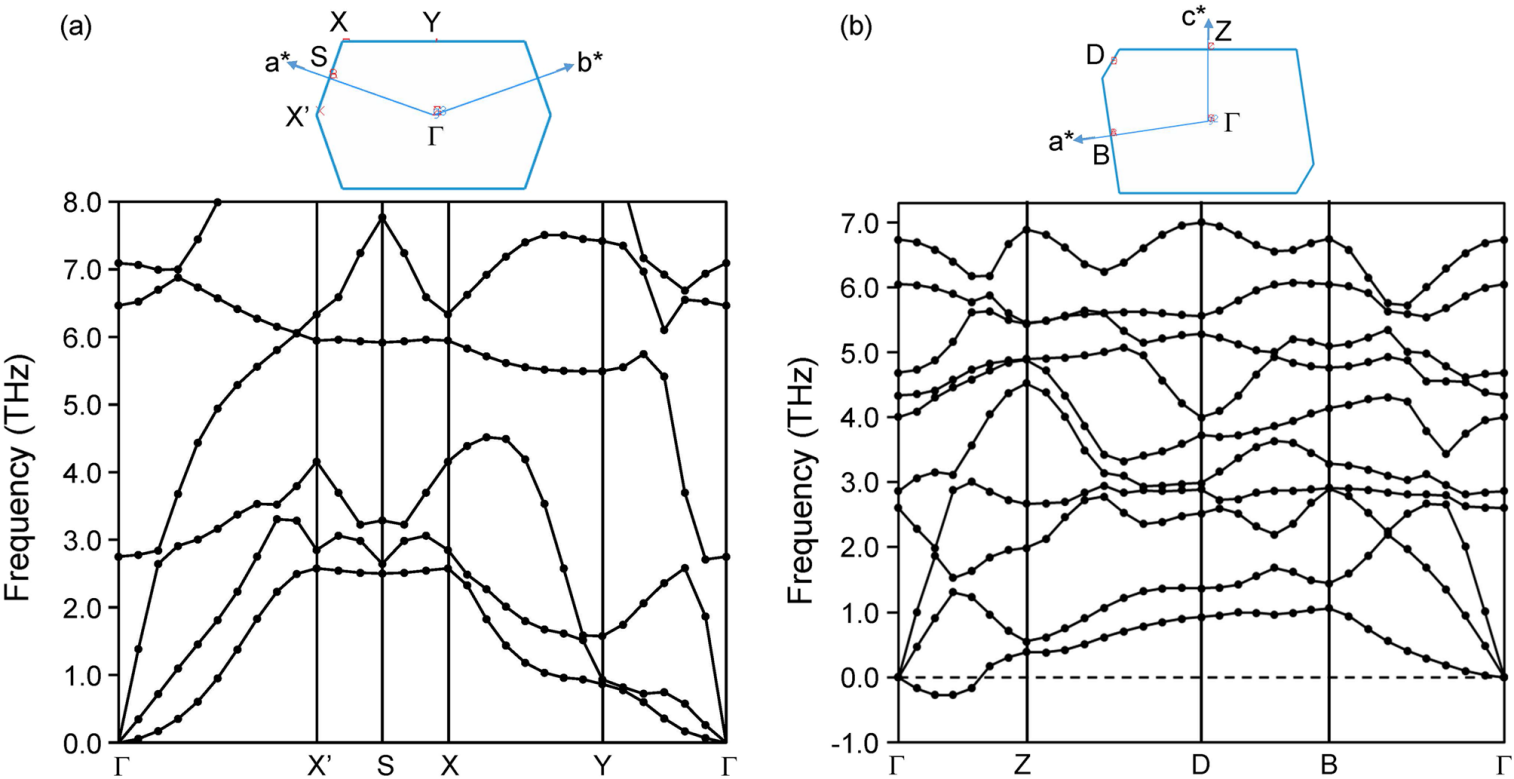
Phonon dispersion curves and BZs of Be-bridged 1D *all-trans* polysilyne chains, **1a** (**a**) and **1b** (**b**). Lattice vectors in the 2D reciprocal space for **1a** and **1b** are represented by (a*, b*) and (a*, c*), respectively.

**Figure 4:**
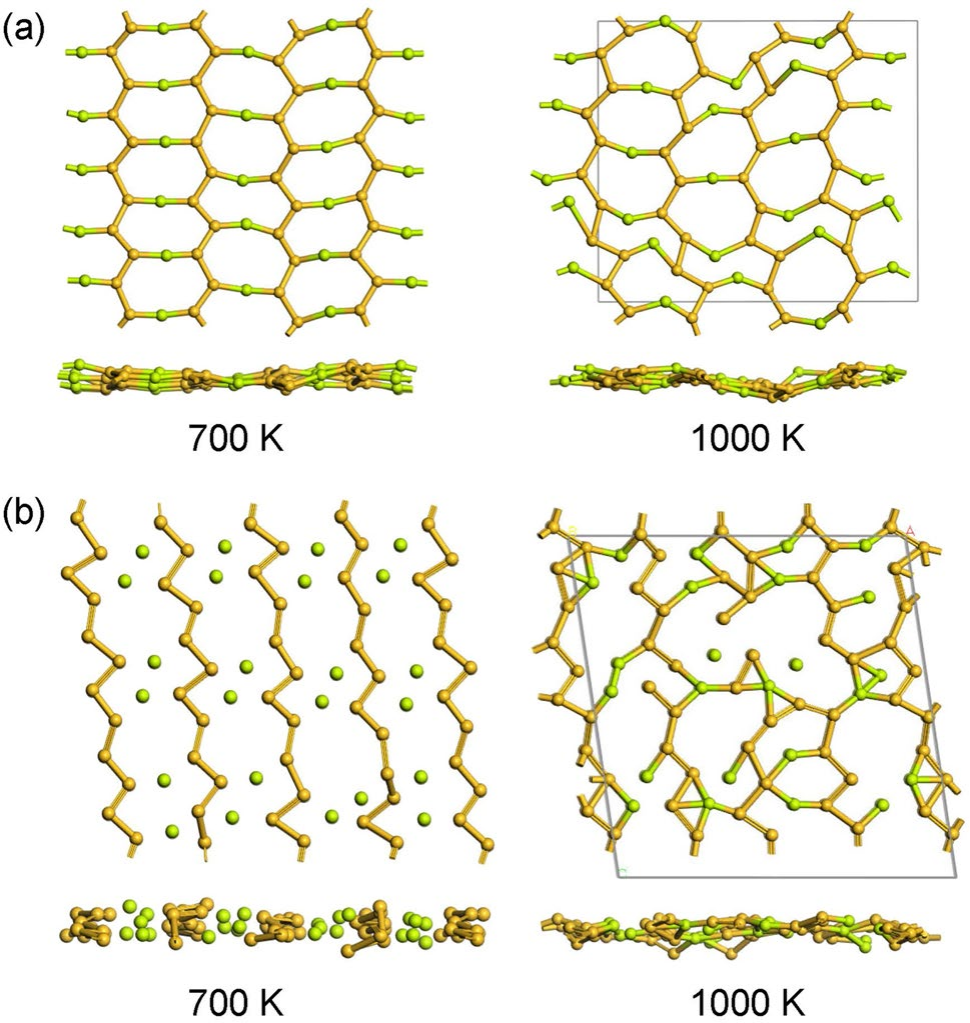
Snapshots of AIMD calculations (top and side views of 2D sheets). (**a**) 5 × 2 × 1 supercell of **1a** conventional cell at 8.0 ps at 700 K and at 1.2 ps at 1000 K. (**b**) 4 × 1 × 3 supercell of **1b** at 8.0 ps at 700 K and at 0.8 ps at 1000 K. Images were created using BIOVIA Materials Studio 2018 (https://www.3ds.com/ja/products-services/biovia/products/molecular-modeling-simulation/biovia-materials-studio/).

We searched for a planar 2D crystal containing 1D *all-trans* polysilyne among various candidate materials with six different even-electron bridging elements from beryllium to sulfur in the second and third rows of the periodic table, and finally reached two different 2D crystals consisting of different 1D *all-trans* polysilynes with beryllium bridged, high-symmetric **1a** and low-symmetric **1b** (Fig. 2). The phonon dispersions throughout the Brillouin zone (BZ) confirm that **1a** is dynamically stable because of the absence of any imaginary frequencies, while **1b** is dynamically unstable due to the existence of imaginary frequencies between the Γ and *Z* points (Fig. 3). Since the magnitude of imaginary value in **1b**, shown as a negative frequency in Fig. 3b, is relatively small, we carefully checked the quality of q-vector set for phonon dispersion calculations at low kinetic energy cutoff (440 eV). The q-vector grid spacing for interpolation in the phonon dispersion calculations with the density functional perturbation theory is specified by the q-point separation parameter, which represents the average distance between the Monkhorst–Pack mesh q-points used in the real space dynamical matrix calculations. The smaller separation parameter corresponds to the denser mesh, giving a more accurate result at the expense of calculation times. A q-vector grid spacing smaller than that used in Fig. 3b (2π × 0.02 Å^–1^) provided a similar phonon dispersion profile near the Γ point to that of the same q-vector grid spacing as in Fig. 3b (2π × 0.03 Å^–1^), and both gave imaginary frequencies between the Γ and *Z* points. Finding the phase transition by using the imaginary frequency mode helps to further explore the stable structure from **1b**^29^. However, the imaginary frequency mode in **1b** is an acoustic translation of the entire crystal, and therefore is not expected to induce a phase transition to a stable structure. Instead, we verified a prerequisite for a viable compound by performing ab-initio molecular dynamics (AIMD) simulations at different temperatures (up to 1300 K). Several snapshots of AIMD calculations are shown in Fig. 4. As seen in Fig. 4, it was found that both **1a** and **1b** are stable during the simulation time up to 8 ps at 700 K. At 1000 K, however, the Si–Si bond cleavage occurs before 1 ps in **1b**, while the Si–Si bond in **1a** is stable at 1 ps, but begins to cleave after 1.5 ps. **1a** is more thermally stable than **1b** and no phase transition from **1a** to **1b** is observed at any stage. The 2D cell area per atom of **1b** is 9.8% smaller than that of **1a**, and the 2D structure of **1b** buckle and wrinkle by heating to cause the cleavage of Si–Si bond. Furthermore, **1a** satisfies the Born–Huang criteria for the mechanical stability of 2D materials: the elastic modulus tensor components C_11_, C_22_, and C_66_ should be positive, and |C_11_ + C_22_| >|2C_12_|. The 2D elastic constants for the conventional rectangle unit cell of **1a** (Fig. 2a) are calculated to be C_11_ = 45 N m^–1^, C_22_ = 78 N m^–1^, C_12_ = 21 N m^–1^, and C_66_ = 1 N m^–1^ with least squares fitting of linear stress versus strain curve on the chosen elastic constants in the strained unit cell^30^, where the stress tensors are calculated using the first-principle calculations.

**Figure 5:**
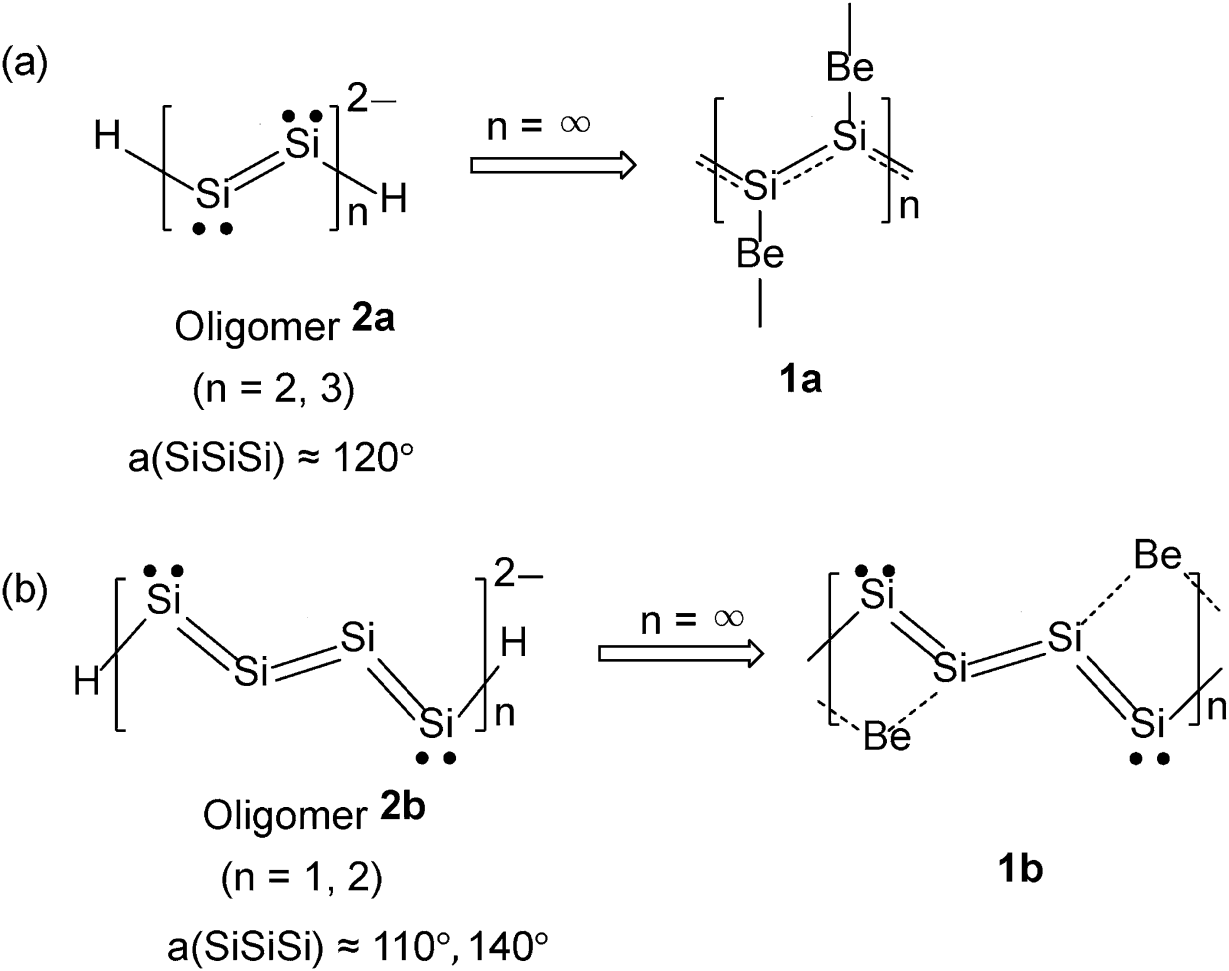
Comparison of polysilyne chains, **1a** (**a**) and **1b** (**b**), with two anionic oligomers, **2a** and **2b** in Ref.^20^, respectively. a(SiSiSi) indicates the Si–Si–Si bond angle of oligomers.

The primitive rhombus unit cell of **1a** with the group symmetry of *D*_*2h*_ contains one beryllium atom and two silicon atoms (Fig. 2a). The short diagonal of rhombus unit cell is parallel to the 1D-polysilyne chain. Two trigonal planar silicons combined by two beryllium atoms construct a distorted honeycomb net reminiscent of that of graphene. Each trigonal planar silicon is connected to two silicon atoms with the bond length of 2.26 Å, which is almost the same as that of single/double bond (2.25 Å in molecular aromatic BeH-terminated hexasilabenzene^23^ and 2.26 Å at the zigzag edge in Be-bridged zigzag silicene nanoribbon^24^). The Si–Si–Si bond angle is 115.7°, as expected for the *sp*^2^ atom type. It is noted that **1a** exhibits no bond alternation along the silicon backbone, suggesting that the π electrons are fully delocalized over the 1D chain (Fig. 5a).

We obtained another crystal **1b** by lowering the group symmetry to *C*_*2h*_ (Fig. 2b). The oblique unit cell with the group symmetry of *C*_*2h*_ contains two beryllium atoms and four silicon atoms. The **1b** is more stable by 0.1 eV atom^–1^ than **1a**. Two of four optimized Si–Si bond lengths take 2.28 Å, while the other two are 2.25 Å and 2.30 Å, respectively. It is noted that the Si–Si bond length of 2.30 Å is definitely longer than the other three (2.25 and 2.28 Å), suggesting a single-bond character as depicted in Fig. 5b. Two of four optimized Si–Si–Si bond angles take a value close to 120°. The other two bond angles are nearly 90°, suggesting the existence of lone pair electrons at the central silicon atoms^20^ (Fig. 5b).

Polysilynes, **1a** and **1b**, are infinite polymers of anionic oligomers **2a** and **2b**^20^, respectively (Fig. 5). The units of **2a** and **2b** consist of two and four silicon atoms, respectively. The bond alternation in oligomer **2a** is reduced in infinite polymer **1a** and gives an almost equal Si–Si bond length, while the basic structure in the unit of oligomer **2b** is retained even in infinite polymer **1b** with two blunt and two relatively sharp Si–Si–Si bond angles with one single and three double Si–Si bonds.

### Electronic and mechanical properties, and thermal conductivity

**Figure 6:**
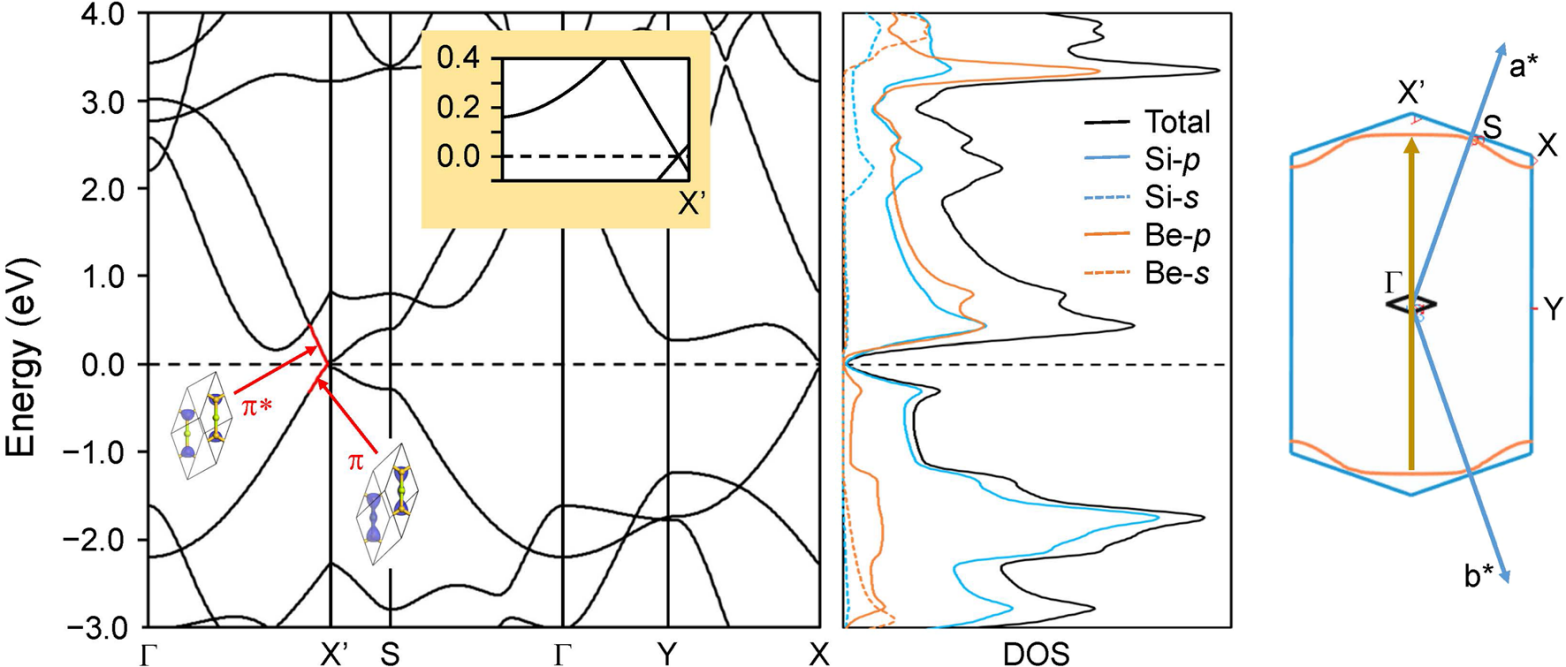
Electronic band structure along high symmetry lines (left), the projected density of states (center), and the Brillouin zone (BZ) (right), for Be-bridged polysilyne chain **1a**. The zero energy corresponds to the Fermi level. Isosurfaces for the molecular orbitals in the unit cell that contribute to the red part of the electronic band dispersion are inserted into the band structure. Inset shows the magnification of the band structure near *X*′ point along Γ–*X*′ line. An equi-energy surface at − 0.286 eV, where a van Hove singularity is located, is overlaid on the BZ (orange solid lines) with the nesting vector connecting two parallel parts (dark orange arrow). Lattice vectors in the 2D BZ are represented by (a*, b*). The rhombus unit cell of real-space 2D crystal is superimposed at Γ point in the BZ (black solid line).

Potential applications of 2D materials are related to their unique electronic properties. In materials science, thermodynamics is not only criterion that guarantees the existence for a given material, but kinetics is also an important key factor. Therefore, we investigated the electronic properties of dynamically stable **1a** (Fig. 6). We found no essential difference in the gross band structure between spin-polarized and spin-unpolarized calculations, suggesting that **1a** is nonmagnetic. The electronic band structure and the projected density of states (DOS) show that **1a** is a Dirac semimetal with linear dispersion in the vicinity of the Fermi energy (Fig. 6) similarly to graphene. But, unlike graphene, the Dirac point is not located at the corner (high-symmetry point) of the BZ, but slightly away from it along the Γ–*X′* line as shown in the inset to Fig. 6. This is due to the lower symmetry of **1a** lattice compared to graphene. The direction of Γ–*X′* line in reciprocal space corresponds to the direction parallel to the 1D chain in real space (i.e. parallel to the short diagonal of rhombus unit cell, see Fig. 6). Two bands which cross each other at the Fermi energy are assigned to the π and π* bands with the out-of-plane 3p_Si_ and 2p_Be_ orbital characters, respectively. The band crossing of π and π* occurs because the two crossed bands belong to different irreducible representations of the crystal symmetry group, one-dimensional Δ_1_ and Δ_2_, respectively, as distinguished by *C*_*2v*_ symmetry around the Γ–*X*′ line. A sizable band gap is expected to open because of the sufficiently strong spin–orbit coupling (SOC) in silicon^24^, but no noticeable gap induced by SOC is observed in **1a**. This is probably due to the Tomonaga–Luttinger liquid nature^31–33^ of the quasi-one-dimensional electron-rich *all-trans* polysilyne chain in which electrons are transferred from the bridging beryllium atoms^19,23^. A remarkable difference in the band structure in comparison with buckled silicene^34,35^ is that the σ–π mixing, which causes a band-gap opening at the crossing point of the σ and π bands, does not occur in **1a** due to the planar structure, like in graphene. It is also noted here that the energy gap at the Γ point is about 2 times smaller than that of graphene. This is probably due to the smaller HOMO–LUMO gap of Si=Si unit than that of C=C.

**Figure 7:**
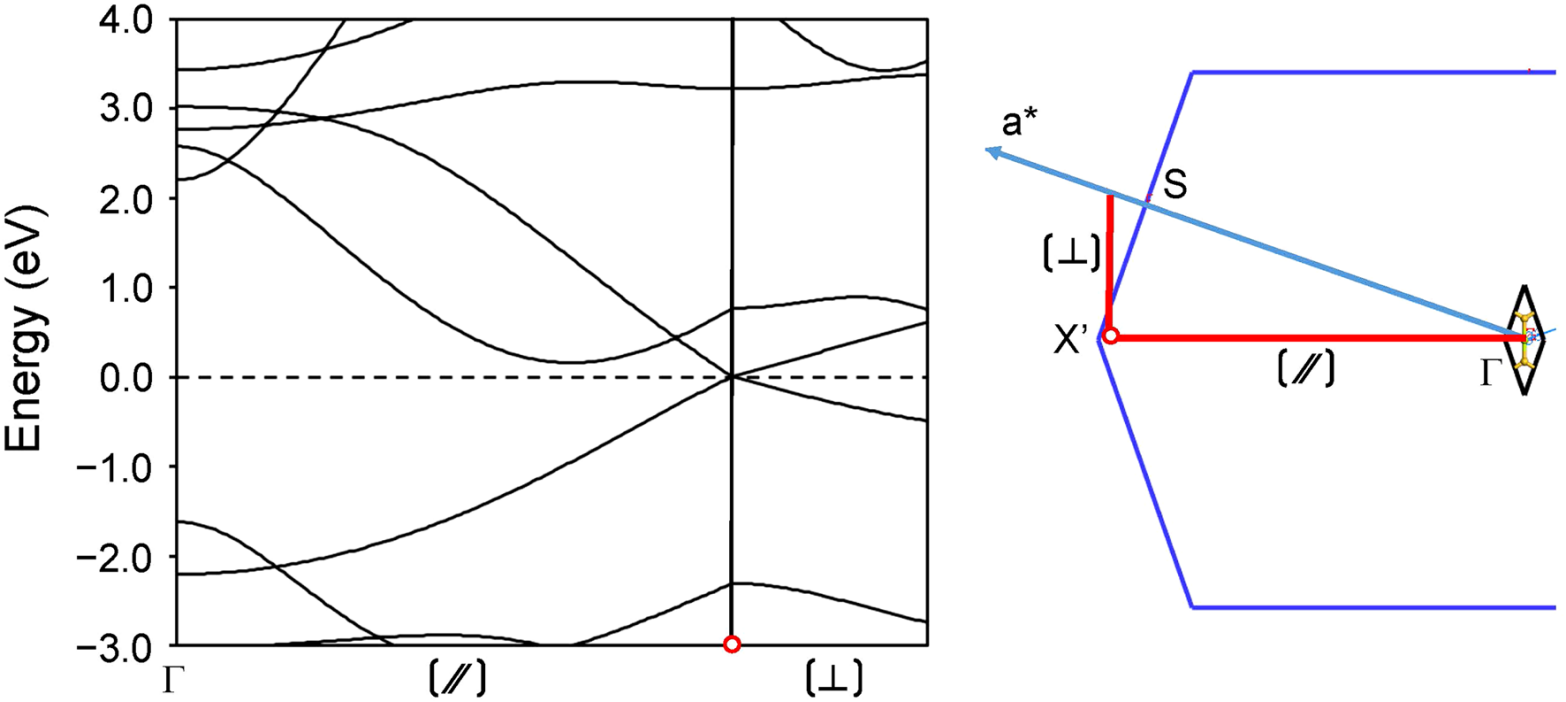
Electronic band structure passing the Dirac point (red open circle) in the direction parallel (//) and perpendicular (⊥) to the 1D chain (red solid lines in the BZ) for Be-bridged polysilyne **1a**. The zero energy of band structure corresponds to the Fermi level. In the BZ, a* indicates the lattice vector in reciprocal space. The rhombus unit cell of real-space 2D crystal is superimposed onto the BZ (black solid line), together with two silicon (gold ball) and one beryllium (green ball) atoms in the unit cell.

Figure 7 shows the electronic band structure passing the Dirac point in the direction parallel and perpendicular to the 1D chain. It is recognized from the behavior of the electron and hole bands that the Dirac cone is categorized into the tilted anisotropic type-I with linear dispersions in all **k** directions, together with a point-like Fermi surface. The anisotropy of Dirac cone is attributed to the 1D nature of the beryllium-bridged zigzag silicon chains of **1a**. The absence of the expected 1D Dirac nodal line^3^ is due to the mixing of the beryllium 2p orbital into the silicon 3p orbital. The Dirac cone is tilted along the Γ–*X*′ direction (parallel direction). The Fermi velocity, defined as *v*(**k**) = (1/ℏ)[*∂E*(**k**)/*∂k*], in the direction from the Dirac point to the Γ point is estimated to be *v*_∕∕_ = 0.5 × 10^6^ m s^–1^, while that to the *X*′ point is 0.7 × 10^6^ m s^–1^. They are 2 and 1.5 times smaller than that of graphene^36,37^, respectively. The Fermi velocity perpendicular to the 1D chain is much smaller (*v*_⊥_ = 0.3 × 10^6^ m s^–1^). Emergence of a tilted anisotropic Dirac dispersion has been predicted in quinoid-type^38^, hydrogenated graphene^39^, and boron allotropes^40,41^, and evidenced in an organic semiconductor α-(BEDT-TTF)_2_I_3_^38,42,43^ and a Bi square net of SrMnBi_2_^44^. Materials possessing this type of dispersions exhibit anisotropic transport properties useful for the valley filtering in p-n junctions^45^ and the generation of photocurrent^46^.

Next, we discuss the difference in the band structure between Be-bridged polysilyne **1a** and graphene in relation to the van Hove singularity. It is known that the DOS in 2D materials is logarithmically divergent at a saddle point (*S* point in Fig. 6) where the van Hove singularity is located^47,48^. At small charge (hole) doping, the Fermi surface with an anisotropic Dirac cone becomes elliptical with the dispersion given by* E*(**k**) = ℏ(*v*_∕∕_^2^k_∕∕_^2^ + *v*_⊥_^2^k_⊥_^2^)^1/2^ for the parallel and perpendicular components of **k** vector (k_∕∕_ and k_⊥_, respectively). At high doping to reach the van Hove singularity located at − 0.286 eV in non-doped **1a**, the Fermi surface is not anymore elliptical, but has parallel lines that are perpendicular to the Γ–*X′* line and extend to the *S* point (Fig. 6). In this case, the Fermi surface is perfectly nested by the wave vector connecting these two parallel parts of the Fermi surface (dark orange arrow in Fig. 6). The most remarkable difference in the band structure between Be-bridged polysilyne **1a** and graphene is that the van Hove singularity is located much closer to the Fermi energy in **1a** than in graphene: the energy interval is 0.3 eV in **1a** while that in graphene is 2 eV. This is due to the smaller energy gap at the Γ point in **1a** than that in graphene. It was recently reported that the Fermi-level shift by 0.5 eV in graphene is achieved by chemical doping^49^, suggesting that the Fermi-level shift by 0.3 eV by chemical doping in **1a** is quite possible. It is also noted here that layered beryllium tetranitride (BeN_4_) was successfully synthesized by the laser-heated diamond anvil technique^2^, suggesting that the same/similar method is applicable for synthesizing beryllium-bridged 1D *all-trans* polysilyne.

**Figure 8:**
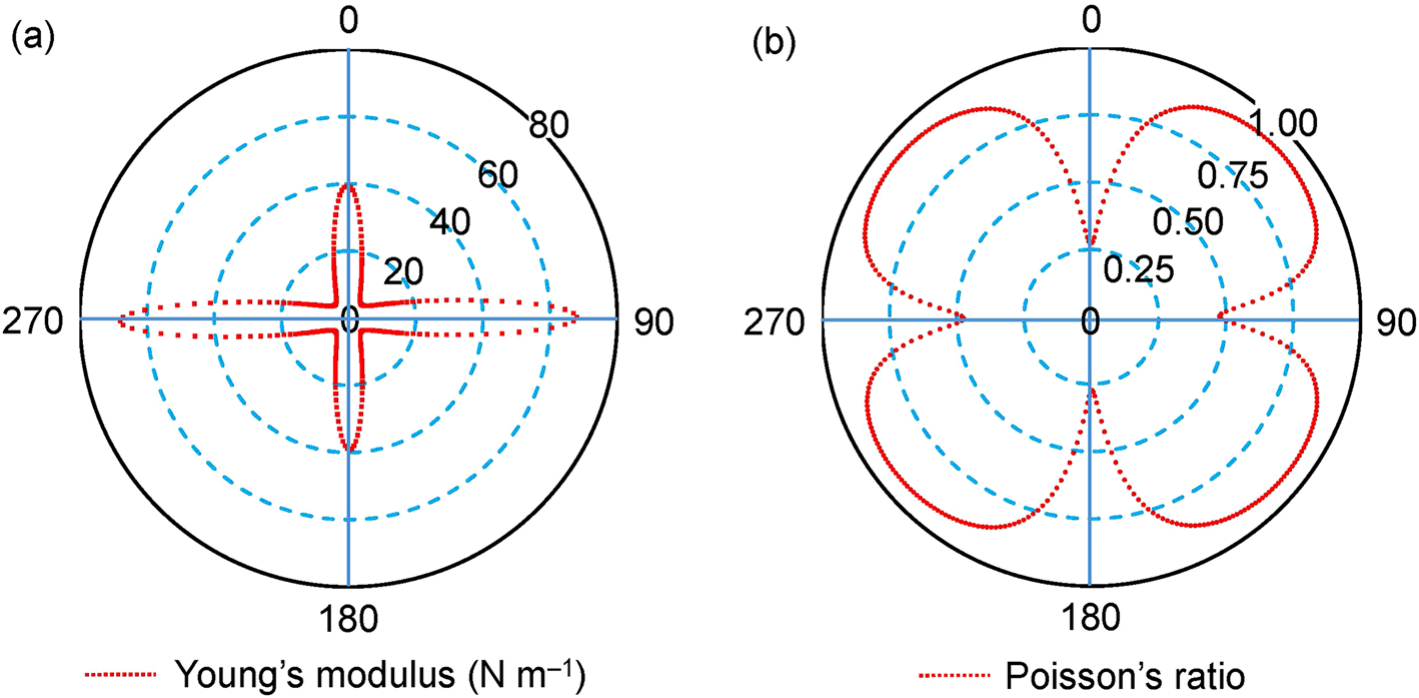
Polar diagrams for the in-plane (**a**) Young’s modulus and (**b**) Poisson’s ratio of **1a**.

The in-plane Young’s modulus *E*(*θ*) and Poisson’s ratio *ν*(*θ*) along an arbitrary direction *θ* (*θ* is the angle relative to the positive direction of 1D polysilyne chain) is expressed as^50–52^

$$E\left( \theta \right) = { }\frac{{C_{11} C_{22} {-}{ }C_{12}^{2} { } }}{{C_{11} s^{4} + C_{22} c^{4} + \left( {\frac{{C_{11} C_{22} {-}{ }C_{12}^{2} }}{{C_{66} }} {-}2C_{12} } \right)c^{2} s^{2} }},$$

$$\nu \left( \theta \right) = { }{-}{ }\frac{{\left( {C_{11} + C_{22} {-}{ }\frac{{C_{11} C_{22} {-} C_{12}^{2} }}{{C_{66} }}} \right)c^{2} s^{2} {-} C_{12} \left( {c^{4} + s^{4} } \right)}}{{C_{11} s^{4} + C_{22} c^{4} + \left( {{ }\frac{{C_{11} C_{22} {-} C_{12}^{2} }}{{C_{66} }} {-}2C_{12} } \right)c^{2} s^{2} }},$$

where *c* = cos *θ* and *s* = sin *θ*. The calculated *E*(*θ*) and *ν*(*θ*) for **1a** are depicted in the polar diagrams in Fig. 8. The diagrams show that both the Young’s modulus and Poisson’s ratio are anisotropic. Young’s moduli parallel and perpendicular to the 1D polysilyne chain, *E*(*0°*) = (C_11_C_22_ − C_12_^2^)/C_22_ and *E*(*90°*) = (C_11_C_22_ − C_12_^2^)/C_11_, are calculated at 39 N m^–1^ and 68 N m^–1^, respectively. These values are close to those of silicene (62 N m^–1^)^53^ and much smaller than those of graphene (335 N m^–1^)^54^. The small in-plane Young’s modulus indicates that **1a** is highly flexible compared to graphene. The Young’s modulus *E*(*90°*) is slightly larger than *E*(*0°*), indicating the stronger bonding perpendicular to the 1D polysilicon chain in the flat monolayered **1a** sheet, which may contribute to the stabilization of the flat conformation of silicon-based 2D sheets.

The heat transfer in 2D material diverges as the distance increases^55^, and the thermal conductivity decreases with increasing temperature^55^. The minimum thermal conductivity *k*_*min*_ at high temperature is an important property for 2D material applications. According to the Clarke model^56^, the minimum thermal conductivity is calculated as:

$$k_{min} = \, 0.{87}k_{B} M_{a}^{{{-}2/3}} E^{1/2} \rho^{1/6} ,$$

where *E* is the Young’s modulus, *ρ* is the density of the crystal structure, *k*_*B*_ is the Boltzmann constant, *M*_*a*_ = [*M*/(*n* × *N*_*a*_)] is the average mass of atoms in the lattice, *M* is the molar mass, *n* is the number of atoms, and *N*_*a*_ is the Avogadro constant. We compare the minimum thermal conductivity of **1a** (*k*_*min,1a*_) with that of graphene (*k*_*min,graphene*_). Our calculations estimate the Young’s modulus of graphene to be 337 N m^–1^ in excellent agreement with the experimentally obtained Young’s modulus of graphene (335 N m^–1^)^54^. The minimum thermal conductivity ratios (*k*_*min,1a*_)/(*k*_*min,graphene*_) are estimated to be 0.22 and 0.28 for the directions parallel and perpendicular to the 1D chain, respectively. Therefore, **1a** is also expected to be a good 2D heat dissipation sheet as good as graphene. The directional dependence of *k*_*min*_ would be related to the strength of bond, i.e. the parallel Si–Si bond vs. the perpendicular Si–Be bond.

## Conclusion

We have proposed 2D silicon-based Dirac materials with a planar configuration consisting of 1D *all-trans* polysilyne by first-principles calculations. One of optimized two 2D crystals (**1a**) with the primitive rhombus unit cell with the group symmetry of *D*_*2h*_ shows no bond alternation along the silicon backbone, suggesting that the π electrons are fully delocalized over the 1D chain. Another crystal (**1b**) is obtained by lowering the group symmetry to *C*_*2h*_ at the oblique unit cell. The crystal **1b** is 0.1 eV atom^−1^ more stable than that of **1a**, but **1b** is dynamically unstable. The band structure calculation has revealed that the higher-symmetric **1a** is a Dirac semimetal with linear dispersion in the vicinity of the Fermi energy and hosts anisotropic Dirac fermions. The Fermi velocity in the direction parallel to the 1D chain is estimated from the tilted anisotropic Dirac cone to be *v*_∕∕_ = 0.5 × 10^6^ and 0.7 × 10^6^ m s^−1^, which is 2 and 1.5 times smaller than that of graphene. We found that 2D beryllium-bridged polysilyne **1a** is a silicon-based air-stable Dirac material, which has a high potential for application to various novel silicon-based electronic devices. The 2D beryllium-bridged polysilyne **1a** is regarded as a promising 2D material with an ultrahigh carrier mobility parallel to the polysilyne chain. The minimum thermal conductivity perpendicular to the polysilyne chain is 30% of that of graphene, indicating a high potential for a good 2D heat dissipation sheet.

## Methods

### Computational details

We performed structural and electronic calculations in the density functional theory (DFT) framework, as implemented in CASTEP code (ver. 2018 and 2019)^57^. The total-energy calculation was based on the plane-wave DFT method within a generalized gradient approximation. The Perdew–Burke–Ernzerhof exchange–correlation functional^58^ was used as implemented in the CASTEP code. Norm-conserving pseudopotentials were used to describe the electron–ion interactions. A fully relativistic norm-conserving pseudopotential was used to examine the effects of spin–orbit coupling. We described the geometric structure under the periodic boundary conditions, adopting a primitive rhombus unit cell for **1a** and an oblique cell for **1b**. To avoid spurious interactions between the periodically repeated replicas, we set vacuum region of 45 Å thickness between the sheets. The valence electron wavefunction was expanded in terms of the plane-wave basis set to a 1200 eV kinetic energy cutoff, unless otherwise noted. The electronic minimization was converged to less than 10^–10^ eV atom^−1^ using a conjugate gradient scheme (the force on each atom was reduced to below 10^−5^ eV Å^−1^). The BZ integration was performed with the Monkhorst–Pack method using k-point mesh with intervals less than 2π × 0.01 Å^−1^. The total energy of the system converged to better than 0.1 meV atom^−1^ for k-point sampling (lower than a chemically sufficient accuracy of 0.01 kcal mol^−1^ (~ 5 K), i.e., 0.4 meV atom^−1^). The geometries were fully optimized for both the cell parameters and atomic coordinates under the constraints of group symmetry of* D*_*2h*_ for **1a** and *C*_*2h*_ for **1b**. The calculation of phonon dispersion was performed using the linear response theory (or density functional perturbation theory)^59^. The convergence criterion for the force constants during a phonon calculation is set to be 10^−5^ eV Å^−2^, and the q-point separation parameters are set to be 2π × 0.04 Å^−1^ for **1a** and 2π × 0.03 Å^−1^ for **1b**, respectively. AIMD simulations in the *NVT* ensemble were carried out for 8 ps with a time step of 1.0 fs to evaluate the thermal stability. The temperature was controlled by using the Nosé–Hoover method^60^. For the estimation of elastic coefficients, the PBEsol functional (a revised PBE functional for solids)^61^, designed to refine PBE for equilibrium properties of bulk solids and their surfaces, was used as implemented in the CASTEP code.

## Data Availability

Data supporting the findings of this work are available within this paper.
